# Evaluating Metabarcoding of Wildflower Associated eDNA to Assess Butterfly Presence and Abundance

**DOI:** 10.1002/ece3.74559

**Published:** 2026-11-11

**Authors:** Cayley Faurot‐Daniels, Diane M. Debinski, Michelle L. Flenniken

**Affiliations:** ^1^ Department of Plant Sciences and Plant Pathology Pollinator Health Center Montana State University Bozeman Montana USA; ^2^ Department of Ecology Pollinator Health Center Montana State University Bozeman Montana USA; ^3^ Pollinator Health Center Montana State University Bozeman Montana USA

**Keywords:** butterflies, environmental DNA, metabarcoding, wildflowers

## Abstract

Rapid and accurate assessment of insect communities is increasingly important in the context of climate change and biodiversity loss. Environmental DNA (eDNA) analysis has proven useful as a noninvasive method for detecting insects in aquatic systems, and methods for terrestrial eDNA sources are being developed. Analysis of wildflower‐associated DNA may be useful for identifying insect pollinators. This study paired traditional butterfly netting surveys in a montane protected area with wildflower‐associated eDNA metabarcoding to test whether the presence and abundance of butterfly species can be accurately assessed. Specifically, Lepidoptera targeting primers were used to amplify two regions of the cytochrome oxidase subunit I (COI) gene from wildflower‐associated eDNA samples. Analysis of the ~350 million sequences obtained identified ~94% as insects, with ~23% assigned to Lepidoptera. Six butterfly taxa, including four at the species level, were identified, including five that were consistent with species captured during netting surveys conducted in parallel. However, butterfly detections from eDNA did not correspond with either contrived or natural flower visitation, and traditional surveys identified 44 butterfly species not captured by eDNA. The most prevalently detected taxa were small‐bodied insects closely associated with flowers. Together our results indicate that additional refinement of protocols and primers is needed before wildflower‐associated eDNA can reliably survey butterfly species.

## Graphical abstract

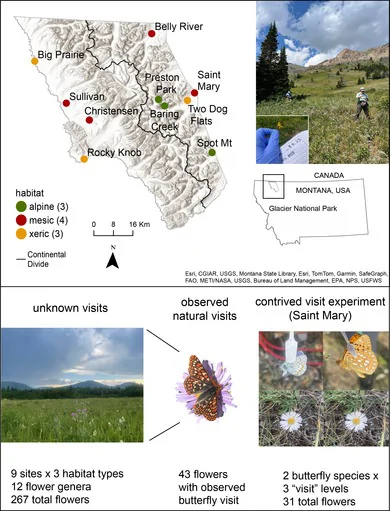

## 1 Introduction

Declines in insect biodiversity and abundance have been reported worldwide (Forister et al. 2010; Dirzo et al. 2014; Raven and Wagner 2021). These losses threaten vital ecosystem services, including pollination, as over 85% of flowering plants depend on animal pollinators (Ollerton et al. 2011). Loss of biodiversity has been noted for nearly a century (Patch 1938; Carson 1962; Soulé 1991; Wilson and Peter 1988; Hughes et al. 1997), though the difficulty in surveying and identification of diverse insect groups continues to hamper efficient monitoring.

Since 2008, rapid advancement of detecting macro‐organisms using trace DNA in the environment, or environmental DNA (eDNA), has enabled characterization of species and communities (Ficetola et al. 2008). Molecular detection methods can detect rare, cryptic or invasive species and provide broad ecosystem assessment using noninvasive techniques, which are becoming increasingly important in the context of climate change and biodiversity loss (Deiner et al. 2017). Environmental DNA research began in freshwater systems and eDNA studies are still dominated by freshwater DNA sources (Beng and Corlett 2020). Applications to terrestrial environments is relatively new, with recent studies leveraging soil (Leempoel et al. 2020; Kirse et al. 2021), spider webs (Gregorič et al. 2022), feces (Kaunisto et al. 2017), pollen (Ronca et al. 2023), air (Roger et al. 2022; Wong et al. 2026) and wildflowers (Thomsen and Sigsgaard 2019; Gamonal Gomez et al. 2023; Harper et al. 2023; Avalos et al. 2024; Coster et al. 2025) as natural aggregates of eDNA to identify arthropods, as well as plants, birds, mammals, and other organisms. Because eDNA deposition and persistence vary across substrates and taxa (Barnes and Turner 2016; van der Heyde et al. 2022), validation with observational data and controlled experiments is still essential, especially for assessing the quantitative potential of eDNA.

Conservation of insect pollinators remains a global priority because of their crucial role in both natural and agricultural systems (Losey and Vaughan 2006; Ollerton et al. 2011; Rader et al. 2016). Studies have shown that DNA collected from flowers can recover diverse insect assemblages, including target pollinators in both wild and agricultural settings, though variation between eDNA collection methods and observational data are also frequently observed (Thomsen and Sigsgaard 2019; Newton et al. 2023; Gamonal Gomez et al. 2023; Harper et al. 2023; Avalos et al. 2024; Coster et al. 2025; Pugh et al. 2025). Consequently, validation studies that pair wildflower eDNA with observational data are needed to understand how reliably eDNA reflects actual flower visitation or community composition.

Butterflies are one of the most studied insect groups due to their conspicuous morphological characteristics and relative ease of capture that aid reliable species identification and community assessment (Debinski et al. 2011; Faurot‐Daniels et al. 2026). Where most insects require a taxonomic expert and a microscope for identification, which is time consuming and costly, the ability to easily capture and identify butterflies in a field setting creates an opportunity to test molecular detections from wildflower eDNA against tandem observational data in a cost‐effective, efficient, and noninvasive manner. Detection of animal taxa from eDNA commonly relies on amplification of a 658‐base pair (bp) region of the mitochondrial encoded *cytochrome oxidase subunit I* (COI) gene, which is commonly referred to as the “barcode” region (Hebert et al. 2003; Lear et al. 2018). Although Lepidoptera (butterflies and moths) are one of the most diverse animal groups, the COI barcode region has been reliably utilized for species identification (Hajibabaei et al. 2006), supported by an extensive reference database (> 10.7 million COI sequences; Barcode of Life Data (BOLD) Systems 2024), including over 1.2 million Lepidopteran records.

Metabarcoding allows many samples to be sequenced simultaneously by incorporating sample‐specific nucleotide tags into primers (Taberlet et al. 2012). Nested metabarcoding further expands throughput by adding a second layer of indexing during sequencing library preparation (Taberlet et al. 2012; Kitson et al. 2019), though care must be taken in experimental design and analysis to account for potential ambiguity created by sequencing errors when reusing indexes in a combinatorial design (Schnell et al. 2015; Guenay‐Greunke et al. 2021). Standardized methods for terrestrial eDNA remain under development (Hassan et al. 2022), and there is considerable variation in all steps including collection, processing, and data analyses (Lear et al. 2018; Ruppert et al. 2019). While many studies report that eDNA techniques either complement or provide higher taxonomic resolution compared to traditional survey methods (Deiner et al. 2017), the potential for quantitative eDNA data requires further investigation. Uncertainty created by variation of eDNA abundance and degradation in different states and environments (Barnes and Turner 2016; Kirtane et al. 2023), the potential taxa‐specific amplification bias of some primer sets (Clarke et al. 2014; Elbrecht and Leese 2015), and variation in bioinformatic processing and analyses of sequencing data (Alberdi et al. 2018; Hakimzadeh et al. 2023), complicate inferences about species presence and abundance. Terrestrial eDNA method validation will require combining observational surveys, controlled experiments, and sequencing data to support ecological inference.

In this study, we leveraged a resurvey of butterfly diversity in Glacier National Park, Montana, using both traditional netting surveys and wildflower‐associated eDNA to assess whether butterfly species presence can be validated from eDNA obtained from wildflowers at a resolution comparable to traditional butterfly netting surveys and if sequence abundance can be correlated with butterfly visitation to flowers (Faurot‐Daniels et al. 2026). Wildflowers with both observed and unknown butterfly visits were collected at monitoring sites following traditional butterfly surveys and a contrived‐visit experiment in which butterflies were placed on flowers in varying numbers of visits was conducted (Figure 1). DNA was isolated from wildflower washes and two sub‐regions of COI were amplified with primer sets established for Lepidoptera: MLepF1/LepR1 (Primer Set 1) and LepF1/MHMR1 (Primer Set 2) (Hajibabaei et al. 2006). Samples were prepared for sequencing using a two‐step PCR nested metabarcoding protocol. To evaluate the quantitative potential of eDNA sequencing, each primer set included a five‐species positive control dilution series, and a subset of wildflower eDNA samples was amplified and sequenced in triplicate. Additionally, because terrestrial eDNA may be especially useful for detecting small or cryptic arthropods, this study provides baseline data on insects associated with wildflowers.

**FIGURE 1:**
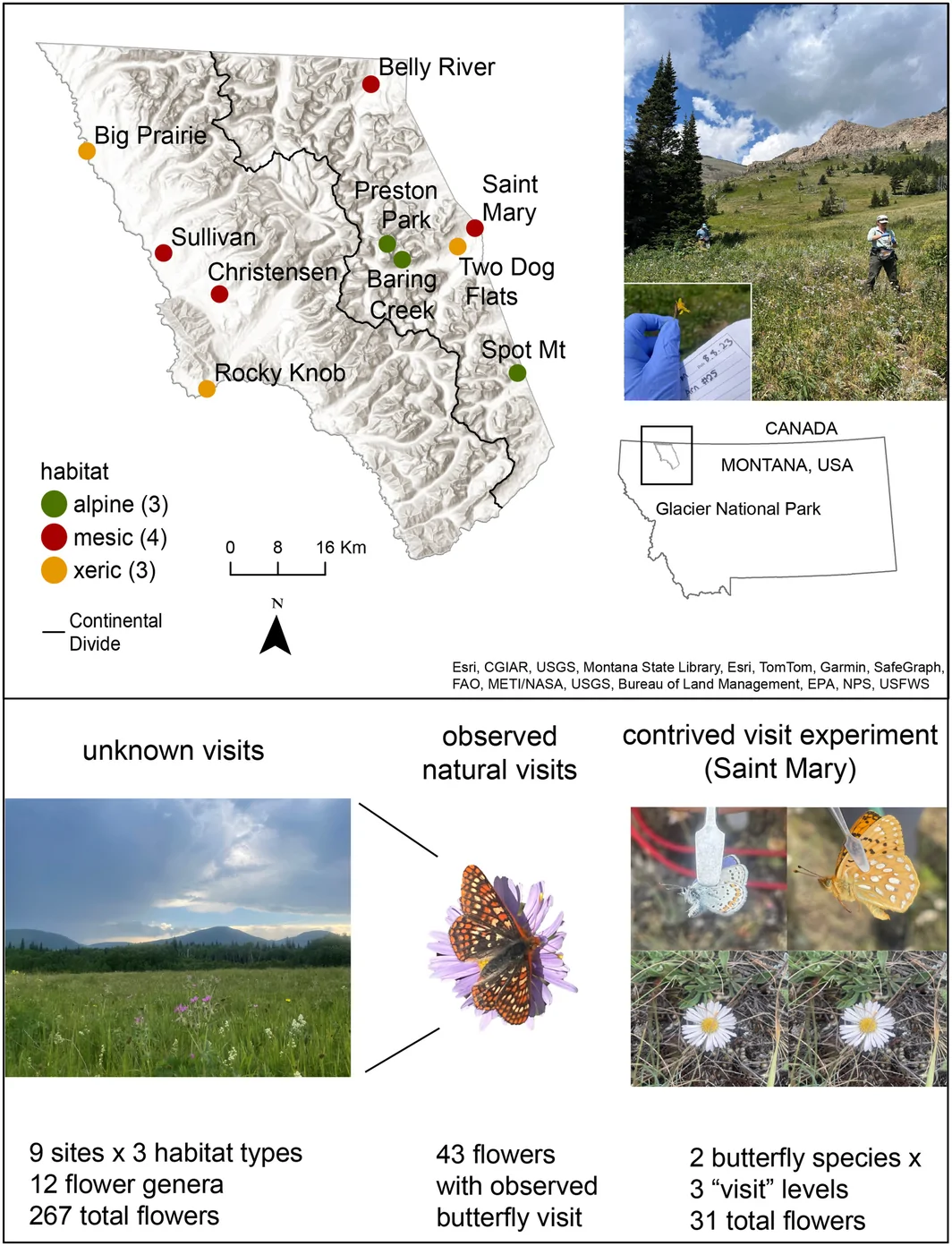
Glacier National Park sample sites and summary of wildflowers for environmental DNA (eDNA) analyses. Wildflowers were sampled at 10 sites representing three habitat types. Twelve flower genera were collected from nine sites for a total of 267 individual wildflowers; 43 had an observed butterfly visit. In addition, 31 wildflowers were collected from a field experiment that involved placement of two butterfly species (i.e., *Speyeria mormonia* and *Plebejus melissa* ) on *Erigeron caespitosus* flowers in a series of contrived visits.

## 2 Materials and Methods

### 2.1 Research Site

Wildflower eDNA samples were collected in Glacier National Park, Montana, U.S.A., across a subset of sites used in an associated butterfly diversity study (Faurot‐Daniels et al. 2026). The larger study surveyed 23 1‐km^2^ sites spanning xeric (low moisture), mesic (moderate moisture), and alpine (above tree line) habitats; from these, nine sites (three per habitat type) were selected for wildflower eDNA collection (Figure 1). At each site, wildflowers were collected from one 50 × 50 m plot, typically the central plot unless flower abundance required using an adjacent plot. A tenth mesic site (St. Mary) was sampled for a controlled butterfly visitation experiment.

### 2.2 Wildflower Collection

Wildflower sampling followed butterfly netting surveys conducted between July 1 and August 16, 2022. Collections were timed to target peak flowering and after at least 2–3 days without precipitation to limit DNA removal from flowers. At each site, 30 inflorescences from two to three dominant, high‐visitation, flower species were collected. Flower collections radiated from the center of the plot and one mature inflorescence was collected with sterile gloves from plants at least 1 m apart from each other. One meter was selected as the minimum distance between flower collections to increase the chances of detecting different butterflies. Flowers with observed butterfly visitation were opportunistically collected, and the butterfly genus, or species, when possible, was recorded. Samples were preserved in silica desiccant and frozen at −20°C upon return from the field. At each collection event, field negative samples (i.e., sterile gauze pad was opened in the field and handled with fresh sterile gloves for 10–15 s) were obtained. In total, 267 flowers from 12 genera were collected across nine sites (Table A1).

### 2.3 Contrived Flower Visits

To confirm butterfly DNA detection from wildflowers and explore whether sequence abundance reflected visitation frequency, a controlled visitation experiment was conducted at St. Mary, Glacier National Park. Two butterfly species common to the study area and well represented in the COI barcode database (Table A2), *Speyeria mormonia* (Mormon Fritillary) and *Plebejus melissa* (Melissa Blue), were placed onto *Erigeron caespitosus* flowers for 1, 5, or 10 consecutive visits (*n* = 5 flowers each, except *S. mormonia* 10‐visit, *n* = 3). One visit was contrived by holding the closed wings of a butterfly with butterfly forceps and physically placing the butterfly's legs on the flower for 5 s. No‐visit controls (*n* = 3) were also collected. Flowers were handled and preserved identically to site collections.

### 2.4 eDNA Extraction and Metabarcoding

Wildflower eDNA samples were processed in a low‐template laboratory at the USGS Northern Rocky Mountain Science Center in Bozeman, Montana, USA. DNA was washed from individual flower heads and extracted using a modified Qiagen DNeasy Blood and Tissue protocol established for arthropod eDNA from flowers (Thomsen and Sigsgaard 2019; Harper et al. 2023); further protocol details are documented in Appendix S2: Supplemental Methods. A DNA extraction negative control (water to extraction column) was included with each extraction round. Two COI regions were amplified using established Lepidoptera‐targeted primer sets, MLepF1/LepR1 (Primer Set 1) and LepF1/MH‐MR1 (Primer Set 2), producing 407‐bp and 311‐bp amplicons, respectively (Hajibabaei et al. 2006). The two amplicon regions overlap across an ~60 bp region of the 658 bp COI barcode (Figure B1).

Contrived and known visit flowers were analyzed individually to validate molecular detections of butterflies associated with a particular flower. The remaining 224 site‐collected flowers did not have a known visit of a butterfly before collection, hereafter referred to as “unknown visit” flowers, and DNA from unknown visit flowers was pooled to maximize samples included in analysis, referred to as “unknown pools” (Table A3). A subset of individual samples was analyzed in three technical replicates. These samples included all 31 contrived‐visit flowers, 25 of the known‐visit wildflowers, and 25 of the pooled “unknown‐visit” flowers. Remaining samples were sequenced in a single replicate (Figure B1).

A two‐step PCR metabarcoding workflow (Figure B2) was used to amplify and index samples (Kitson et al. 2019; Guenay‐Greunke et al. 2021). In the first PCR (PCR1), 12 forward and eight reverse primers with unique internal indices were used to amplify the COI target regions and tag resulting PCR products (Table A4). To facilitate evaluation of index hopping after sequencing, 12 tag combinations were not used in this study. Products from PCR1 (84 reactions for each of nine PCR plates) were pooled, purified, size‐selected with magnetic beads, and used as template in the second PCR (PCR2), which added Illumina sequencing adapters and unique dual indices. The resulting sub‐libraries were pooled into a final sequencing library following bead‐based cleanup and Bioanalyzer quantification (Table A5).

All controls were sequenced, including field negatives (sterile gauze), extraction blanks, no‐template PCR controls, and PCR positive controls consisting of mixed insect DNA from five species: *Bombus rufocinctus* (red‐belted bumble bee), *Apis mellifera* (honey bee), *Phymata americana* (jagged ambush bug), *Coccinella transversoguttata* (transverse lady beetle), and *Pieris rapae* (cabbage white butterfly) (Figure B3). To assess whether sequence abundance could reflect DNA input, a dilution series of the positive control (5.0, 2.5, 1.25, and 0.625 ng/ul) was also amplified both alone and in a background of mixed wildflower eDNA representing genera of manipulated and known‐visit wildflowers (*Arnica, Erigeron, Potentilla, Senecio, Solidago*).

### 2.5 High Throughput Sequencing, Bioinformatic Processing and Taxonomic Assignment

The final wildflower‐associated eDNA library was sequenced (2 × 250 bp) on an Illumina NovaSeq SP flowcell at the University of Illinois Roy J. Carver Biotechnology Center. Reads were de‐multiplexed using the outer indices and then by internal indices to associate sequences from individual or pooled wildflowers (National Center for Biotechnology Information (NCBI) Sequence Read Archive (SRA) (BioProject PRJNA1422262), Dryad dataset: Lepidoptera COI targeted eDNA from wildflowers, doi: 10.5061/dryad.rv15dv4nv). Computation efforts were performed on the Tempest High Performance Computing System, operated and supported by University Information Technology Research Cyberinfrastructure at Montana State University.

Index hopping rates were calculated by comparing unexpected tag combinations from the 12 unused primer index combinations to those that were expected (Schnell et al. 2015; Guenay‐Greunke et al. 2021). The DADA2 bioinformatic pipeline (Callahan et al. 2016) was then implemented via the Targeted Amplicon Diversity Analysis (TADA) workflow in Nextflow, which includes quality filtering, sample denoising, read merging, and chimera removal (Lennard et al. 2020). Outputs of the pipeline were amplicon sequence variants (ASVs), or sequences that vary by one or more nucleotides.

ASVs were taxonomically assigned by global alignment (VSEARCH in QIIME2) to two COI reference databases derived from the Barcode of Life Database (BOLD): (1) a broad US/Canada animal barcode database and (2) a filtered database of butterfly species expected in Glacier National Park. A 95% similarity threshold was used for assignment based on empirical sequence similarity among regional butterfly COI records (Appendix S2). Assignments were imported into R (R Core Team 2023) for downstream filtering and analysis.

### 2.6 Sequence Filtering and Analyses

Sequences corresponding to contaminants were identified using decontam (Davis et al. 2018) and removed along with ASVs represented by fewer than five sequences or sequences substantially shorter (i.e., < 100 bp) than expected amplicon sizes; 407‐bp for Primer Set 1 and 311‐bp for Primer Set 2 (Tables A6 and A7). Positive‐control species detected in field or laboratory negatives were excluded from wildflower samples.

To assess continuity between technical replicates of each primer set, accumulation curves of ASVs and unique taxa were created using vegan (Oksanen et al. 2022). Analyses of observed richness (number of unique taxonomic identifications) or detections (sum of presence values) by taxonomic rankings were conducted using presence or absence values across all noncontrol samples, where samples with technical replicates were merged so that a “1” was given if an ASV was present in any of the three replicates and “0” if the ASV was absent from all three technical replicates. Comparisons of taxonomic richness of the same samples between both primer sets were tested using paired Wilcoxon ranked sign tests. There was not enough data to statistically compare butterfly detections between traditional and molecular methods. As such, our results are primarily descriptive.

## 3 Results

### 3.1 Wildflower‐Associated eDNA Samples and Sequencing

To assess whether butterflies could be detected, and possibly quantified, from wildflower‐associated eDNA, 298 wildflowers were collected from 10 sites in Glacier National Park within an hour of butterfly netting surveys. Of these, 74 flowers had known butterfly contact and were retained as individual samples; the remaining 224 flowers were pooled by site and genus (Figure 1, Table A3). A dilution series of a five‐species positive control was included to evaluate quantitative potential (Figure B1). Wildflower‐associated DNA was amplified with two COI primer sets. The nested metabarcoding workflow generated nine sub‐libraries comprising 710 tagged PCR reactions, including all samples and controls (Figure B2). Illumina NovaSeq sequencing produced > 800 million paired‐end sequences, with ~350 million sequences retained after filtering. Estimated index hopping was low for sub‐libraries containing wildflower samples (0.008%–0.822%) and higher for the positive‐control plate (9.5%, Table A8).

Primer Set 1 (PS1) yielded ~223 million retained sequences, and Primer Set 2 (PS2) yielded ~124 million (M). Mean sequences per sample were 524,030 for PS1 (range 0–4.29 M) and 381,296 for PS2 (range 0–9.46 M) (Table A9). Low‐yield samples (< 10 sequences) were uncommon for PS1 (6 samples) but more frequent for PS2 (49 samples), largely from plate 8, which had low product yield after cleanup. Most low‐yield samples were pooled eDNA samples lacking observed visitation; only two were shared between primer sets (Table A10).

### 3.2 Mixed Insect DNA Dilution Series and Sequencing Summary

Three out of five expected positive control species were identified using PS1: *Phymata americana, Coccinella transversoguttata*, and *Pieris rapae*. The majority, 97.12% to 99.78%, of the dilution series sequences were identified as *Phymata americana* . As expected, *Pieris rapae* was the only species identified using PS2 (Figure B3). Positive control insect DNA was also amplified in floral DNA backgrounds that included five plant genera: *Arnica, Erigeron, Potentilla, Senecio*, and *Solidago*. Amplification of *Pieris* in mixed floral backgrounds varied widely (10%–90% of total reads depending on flower genus; Table A11). Dilutions with lower amounts of DNA (i.e., 1.25 ng/ul and 0.625 ng/ul) failed to amplify with PS2. Because species proportions were highly uneven and did not track DNA input, sequence abundance was not considered quantitative, and downstream analyses used presence–absence.

Positive‐control species were also detected in some negative controls, although overall sequence counts in negative controls were low (Table A9). Field negatives contained up to 14,650 reads with PS1 (mean 2225) and 19,488 with PS2 (mean 2223); elevated samples were dominated by arthropod sequences unassigned to any taxonomic order. Amplifications from buffer‐only negative controls were typically low using PS2 (mean 11 reads) and higher for PS1 (mean 5187), though two of 14 extractions contributed most of these sequences and the majority were assigned to *Phymata americana* . The number of sequences obtained from no‐template control (NTC) samples was low for both primer sets (PS1 mean: 131; PS2 mean: 1142); the majority of sequences associated with NTCs for PS2 were assigned to *Pieris rapae* (Figure B4). Because control species were detected in negatives and were native to the study region, all positive‐control taxa were removed from further analyses.

### 3.3 Technical Replicates Improve Taxon Recovery

Technical replicates produced broadly consistent ASV and taxon counts across plates for both primer sets (Figure B5). For PS1, the three individual replicates of 81 priority samples produced 2357–2771 ASVs and 95–107 taxa; combining all three replicates increased totals to 4768 ASVs and 142 taxa. For PS2, individual replicates yielded 907–976 ASVs and 42–47 taxa, increasing to 2075 ASVs and 63 taxa when all three were combined. These patterns support that technical replication increases total taxon recovery, particularly for rare taxa.

### 3.4 Butterfly eDNA After Controlled Visitation Experiments

Assessment of sequences associated with a controlled visitation experiment, where two butterfly species were placed on flowers in one, five, or 10 “visits”, revealed that no sequences were identified to either of the two target species ( *Speyeria mormonia* or *Plebejus melissa* ). Although PS2 generated 59 million Lepidopteran sequences from 401 ASVs and 800,000 Lepidopteran sequences from 53 ASVs were generated with PS1, all Lepidopteran ASVs shared ≤ 88% nucleotide identity with barcode records from these species, confirming the absence of true matches (Table A12). Thus, contrived visits did not yield detectable target‐species eDNA, and sequence abundance did not correlate with visit numbers.

### 3.5 Lepidoptera Detections Across All Wildflower eDNA Samples

Across all insect detections, Lepidoptera represented 3.4% of PS1 detections (415 of 12,041) and 29.5% of PS2 detections (995 of 3368; Figure 2). A majority of samples had more than 1 Lepidoptera ASV (PS1: 101/145 samples; PS2: 81/145). Mean Lepidoptera ASVs per sample were 2.9 for PS1 and 6.9 for PS2, though the number of Lepidoptera ASVs per sample did not differ significantly between primer sets (paired Wilcoxon V = 3045.5, *p* = 0.73).

**FIGURE 2:**
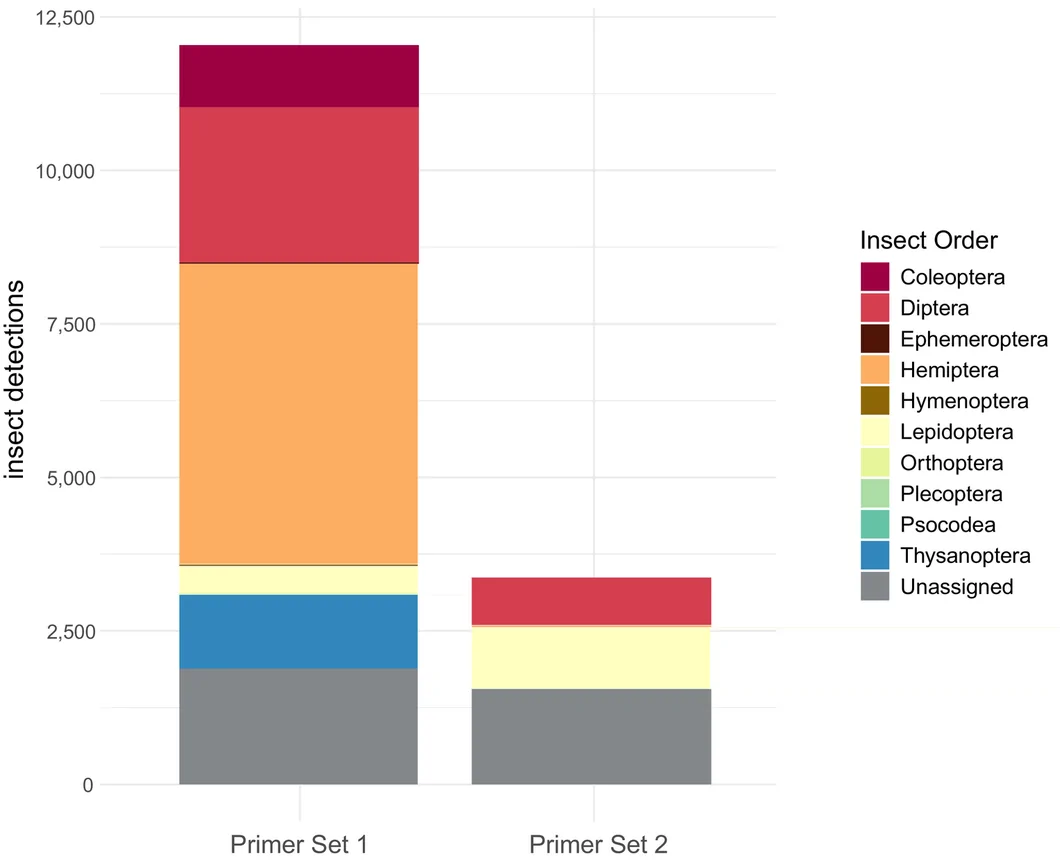
Insect detections varied with *cytochrome c oxidase I* (COI) barcode primer sets. Primer Set 1 resulted in 12,200 total detections and Primer Set 2 (LepF1/MHMR1) resulted in ~3000, which were assigned to 10 insect orders. Insect ASVs not identified to order are represented by “Unassigned” (grey).

Across both primer sets, 19 Lepidoptera families were detected; eight were shared, four were unique to PS1, and 7 were unique to PS2 (Figure 3). Four of the five butterfly families known from Glacier National Park were detected; Hesperiidae (Skippers) were identified with both primer sets, Nymphalidae (Brush‐footed butterflies) were only detected with PS1, and Lycaenidae (Blues) and Papilionidae (Swallowtails) were only detected with PS2. No Pieridae (Whites and Sulphurs) detections remained after removing ASVs identified to *Pieris rapae*.

**FIGURE 3:**
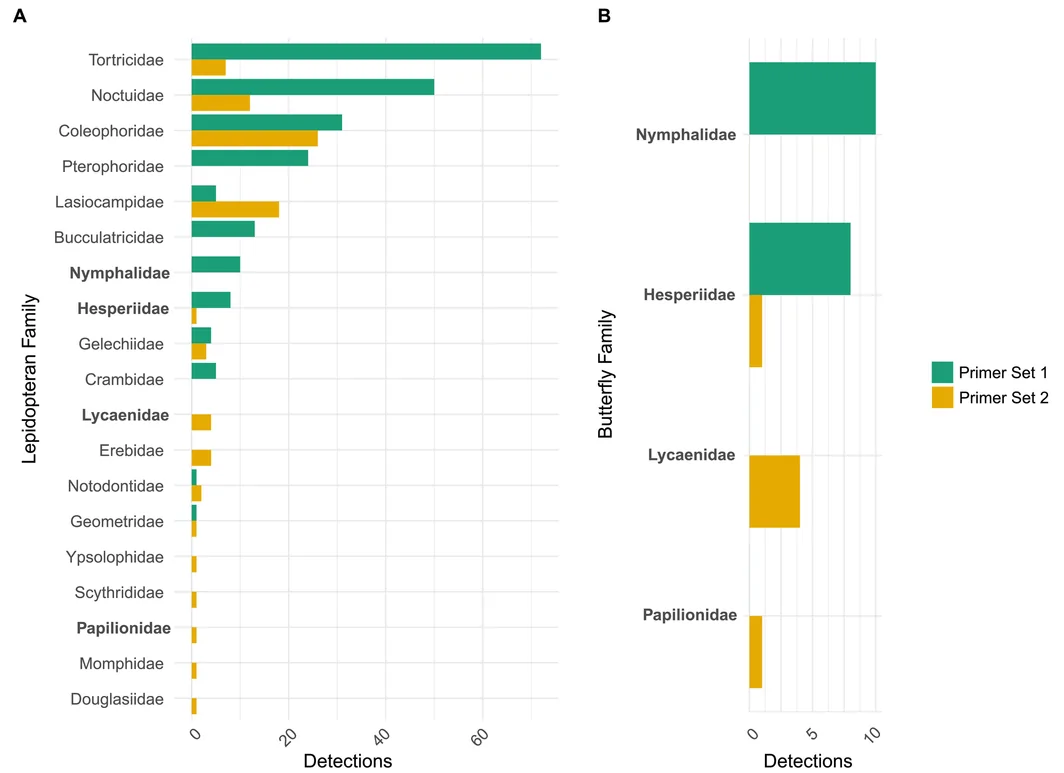
Lepidopteran detections varied by primer set. (A) 19 Lepidopteran families were identified using both primer sets (Set 1‐green and Set 2‐yellow). Butterfly families are highlighted in bold and are subset in panel (B). In total, four butterfly families were detected using both primer sets. Primer Set 1 detected Nymphalidae and the majority of Hesperiidae detections (i.e., 8 of 9); Lycaenidae and Papilionidae were only detected by Primer Set 2.

### 3.6 Comparison of eDNA Butterfly Detections With Visitation and Netting Data

Across 74 individual flowers with known visitation and 71 pooled samples representing 224 flowers, six butterfly taxa (four species, two genera) were detected, totaling 24 detections across 21 samples from four sites (Figure B6). *Oarisma garita* (Garita Skipperling) was the only species detected by both primer sets.

Traditional netting surveys that were conducted before wildflower eDNA collection detected 47 species from five families, whereas eDNA detected six taxa from four families (Figure 4). Five of six eDNA detections matched known site occupancy (e.g., *Cercyonis pegala* at St. Mary; *Thymelicus lineola* , *Phyciodes*, and *Tharsalea* at Spot Mt., Preston Park, or Christensen). No butterfly eDNA detections matched the 43 flowers with direct natural visitation observations (Table A13).

**FIGURE 4:**
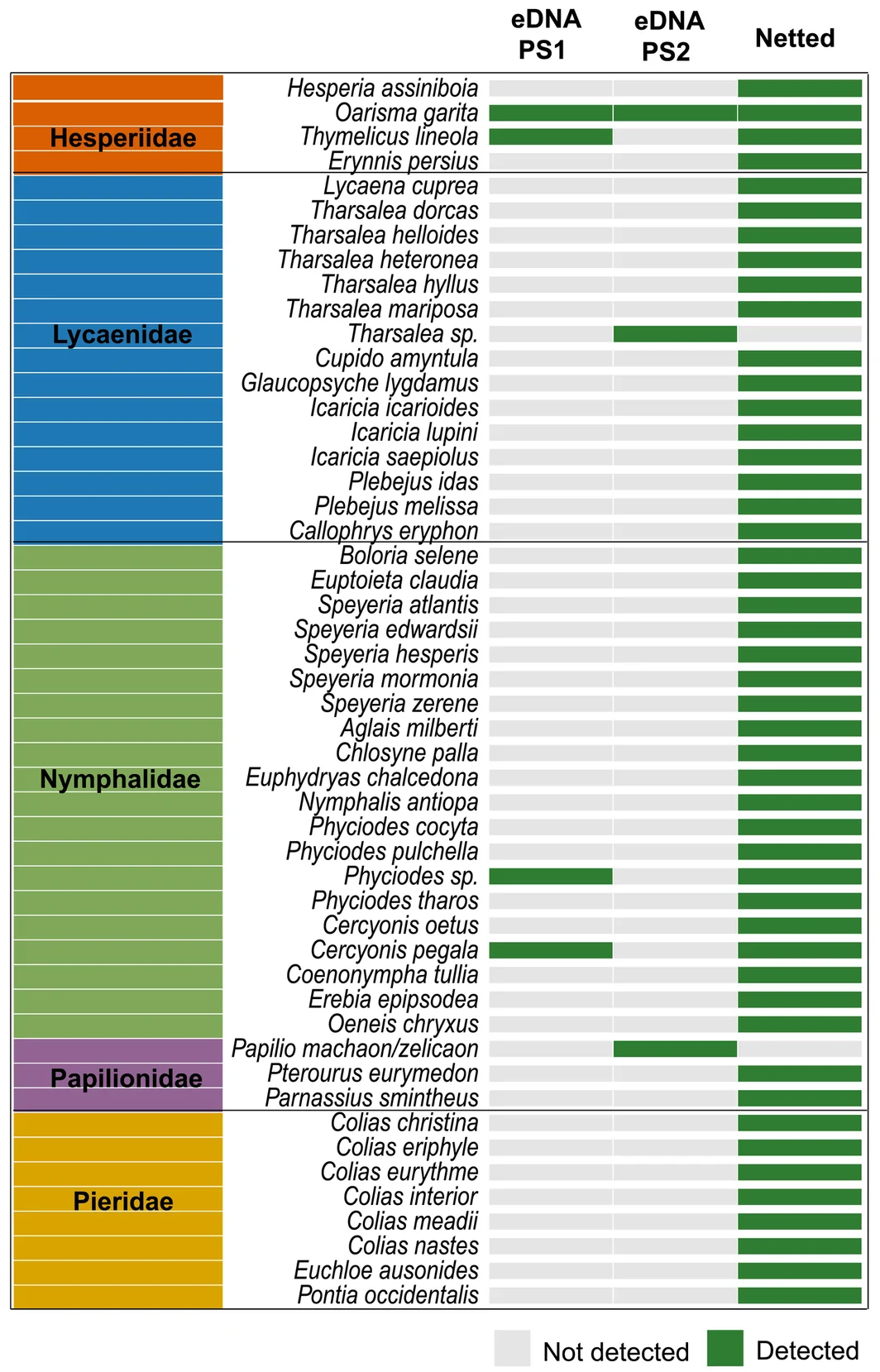
Species level comparison of butterfly detections between traditional and molecular methods. Traditional netting surveys identified 47 butterfly species while six butterfly taxa, four to species and two to genus, were identified from eDNA using two primer sets (PS1 and PS2). Only one species, *Oarisma garita*, was identified by both primer sets and netting surveys. One species was detected by eDNA only, *Papilio machaon/zelicaon*, which was resolved to two different species using separate COI databases, a regional North American database and local Glacier National Park database, respectively. Species names match the Barcode of Life Database (BOLD) as they were downloaded in March of 2023 and may not reflect recent taxonomic revisions.

Using a Glacier‐specific COI reference database (Appendix S2) did not increase detections; the only difference was a shift in assignment for one *Papilio* ASV from *P. machaon* (broad database) to *P. zelicaon* (GNP‐specific), which was confirmed as locally present in 2022 surveys but not captured on the day of wildflower collection.

### 3.7 Non‐Target Insect Detections

Insects other than Lepidoptera were identified with both primer sets (Figure 2). With PS1, Hemiptera (true bugs) and Diptera (flies) dominated detections, with 41% and 30%, respectively. Thysanoptera (thrips) and Coleoptera (beetles) comprised 10% and 8% of detections, respectively, while 3% were Lepidoptera. Remaining orders detected with PS1 were less than 1% of detections each, and included Hymenoptera (bees, wasps, ants), Orthoptera (grasshoppers, crickets), Ephemeroptera (mayflies), and Psocodea (book and bark lice). PS2 produced fewer total detections but proportionally more Lepidoptera (30%). Other orders detected were Diptera (23%), Hemiptera (1%), and less than 1% for Coleoptera, Hymenoptera, Plecoptera (stoneflies), and Thysanoptera. Unassigned insect ASVs made up 15.7% of PS1 detections and 46.1% of PS2 detections.

The most common families across both primer sets were Miridae (leaf or plant bugs), Tephritidae (fruit flies), and Chironomidae (midges); other dominant families differed between primer sets (Table 1).

**TABLE 1:** Description of the 10 most detected insect families by primer set.

Family	Common names	Usual size	Where typically found	Life history/ecology notes	*N* Set 1	*N* Set 2	Total *N*
Miridae	Leaf or Plant Bugs	< 10 mm	Vegetation	Phytophagous	1700	38	1738
Tephritidae	Fruit Flies	2–25 mm	Flowers, vegetation	Phytophagous, many oviposit on flower heads of *Asteraceae*	738	565	1303
Chironomidae	Midges	1–10 mm	In or near freshwater	Adults congregate in large swarms near freshwater	434	53	487
Curculionidae	Snout Beetles	1–35 mm	Vegetation	Phytophagous	305	1	306
Chrysomelidae	Leaf Beetles	< 12 mm	Flowers, vegetation	Phytophagous	164	0	164
Thripidae	Common Thrips	< 1.5 mm	Flowers, vegetation	Phytophagous	124	1	125
Anthomyiidae	Anthomyid Flies	3–12 mm	Flowers, dung	Adults are nectar and pollen feeders	96	18	114
Delphacidae	Delphacid Plant Hoppers	1.5–10 mm	Mainly grass feeders	Common, mostly grass feeders	106	0	106
Rhagionidae	Snipe Flies	8–15 mm	Wooded areas, dense vegetation	Common	69	23	92
Tortricidae	Tortricid/Leafroller Moths	8–35 mm	Vegetation	Common, larvae are leaf rollers/leaf tiers may feed on flowers	72	7	79
Cecidomyiidae	Gall Gnats	< 3 mm	Vegetation	Most larvae are gall makers, some plant feeders	73	1	74
Coleophoridae	Casebearer Moths	5–26 mm	Vegetation	Larvae are leaf miners, may use flowers larval cases	31	26	57
Chloropidae	Frit Flies	Small to very small	Vegetation	Common, larvae typically grass feeders	16	31	47

## 4 Discussion

Wildflower‐associated eDNA can serve as a source of DNA for insect monitoring. In this study, the majority of sequences obtained were identified as insects, and Lepidoptera comprised a substantial fraction of those detections. However, butterflies were generally not detected in accordance with known or contrived visitation, and sequence abundance did not correlate with insect DNA input of positive controls. These findings indicate that additional methodological development is needed before wildflower‐associated eDNA can reliably assess butterfly communities in the field.

### 4.1 Limited Detection and Importance of Technical Replicates

The nested metabarcoding strategy allowed high‐throughput sequencing of hundreds of PCR reactions in parallel (Figure B2) and yielded high‐quality insect sequence data. Estimates of index hopping in this sample cohort were low (Table A8) and most low‐abundance samples were likely due to low library yield rather than methodology. Despite obtaining large numbers of insect sequences, positive control tests indicated that sequence abundance could not be used to infer relative abundance of taxa. One species ( *Phymata americana* ) consistently comprised over 90% of total sequences in the five species positive control with PS1, and the proportion of *Pieris rapae* sequences varied in different floral backgrounds with PS2 (Table A11). Bias introduced by variable priming efficiency among taxa, where taxa that have fewer mismatches in a primer region may amplify more efficiently, has been reported as a strong factor complicating abundance estimates (Piñol et al. 2015; Krehenwinkel et al. 2017). However, this does not explain the overabundance of *Phymata americana* for PS1 as *P. americana* and *Pieris rapae* both had one mismatch on the forward primer and two mismatches on the reverse primer (Figure B3). Additional experiments of positive control dilutions in varying concentrations of background DNA will advance the understanding of how sequence quantification varies from different sample origins.

Technical replicates improved detection of rare taxa; combining two or three replicates increased the number of ASVs and unique taxa identified. Several studies have found more variation between replicates for low abundance samples (Leray and Knowlton 2017; Shirazi et al. 2021). Many wildflower‐associated eDNA samples in this study were of low abundance, particularly for PS2, so it is perhaps unsurprising that PCR replicates were inconsistent (Table A10). Indeed, accumulation curves for both primer sets did not reach visible asymptotes indicating more unique taxa would be found with additional replicates (Figure B5). Because butterflies were of particular interest to this study and capturing both rare and abundant species in sequencing data would be critical to comparing detections to traditional surveys, low abundance samples were retained and detections from all three technical replicates were considered. While this approach may be less stringent than those that only consider taxa that are present in at least two or even three technical replicates (Alberdi et al. 2018), tandem observational and experimental data of butterfly presence and known contact with specific flowers allowed for butterfly detections from wildflower‐associated eDNA sequencing to be validated. These findings reinforce the value of replicates in studies targeting rare species and care must be taken when applying stringent presence thresholds.

### 4.2 Primer‐Specific Detection of Lepidoptera

Both COI primer sets detected Lepidopteran taxa, though the composition of families varied (Figure 3). While order‐level richness was similar between primer sets, certain butterfly families were only detected with one primer set. The two sub regions amplified with both primer sets cover the full 658‐bp COI barcode region with ~60‐bp of overlap but yielded very different results between the forward half (PS2: LepF1/MHMR1—310 bp) and the latter half (PS1: MLepF1/LepR1—407 bp) of the barcode region. These results suggest that multiple primer sets may be required to comprehensively characterize butterfly communities from eDNA.

### 4.3 Limited but Credible Butterfly Detections

Butterflies were detected in only a small fraction of wildflower‐associated eDNA samples and target taxa were not identified from flowers with observed butterfly visits. Among all wildflower eDNA samples, six butterfly taxa were identified across four of the 10 sampled sites, with five taxa validated by traditional netting surveys (Table A13). One taxon identified only by eDNA was resolved to a local species when using a regional reference database, highlighting the importance of reference data. All but one wildflower eDNA sample that generated identifications of butterfly taxa were sequenced in three technical replicates, further supporting the importance of replicates for detecting rare taxa (Figure B6). Traditional netting surveys conducted on the same day as eDNA sampling identified an additional 44 butterfly species (Figure 4). No species of the family Pieridae were identified by eDNA, after removing *Pieris rapae* sequences as likely spillover from positive controls, whereas eight Pierid species were detected with netting surveys. Given that there were so few butterfly species detections via eDNA overall, chance may explain the absence of Pierid species detections via eDNA. Pierids are frequent flower visitors (Brock and Kaufmann 2003) and identifications of *Pieris rapae* in positive controls indicated a member of the Pierid family could be identified with both primer sets (Table A11). More investigation would be needed to speculate fully on why Pierid species were not detected by eDNA.

We did not expect to detect eDNA from butterfly larvae in this study since many of the butterfly species in Glacier National Park are univoltine, so larval and adult butterflies are not present at the same time. Of the six taxa that were detected via eDNA, only two use flowering plants as larval hosts, while the others use grasses (Shirey et al. 2022). Moreover, some flower‐associated species feed on the leaves rather than the flowers (pers. obs.), reducing the likelihood of detecting their DNA on flowers.

Butterflies were not detected from wildflowers with known or contrived visits (Table A13). It is unlikely that the lack of butterfly detections is due to the resolution of the reference database. Butterflies are well represented in the COI barcode database; from a list of 126 species either observed by this study or by citizen scientists (iNaturalist) in Glacier National Park, only six species have fewer than 10 records, and none have fewer than five records (Table A2). The primers used in this study were established in their ability to distinguish Lepidopteran species, and the flanking primers for each primer set (LepF1 and LepR1) are often used to create COI barcode references (Hajibabaei et al. 2006). Therefore, additional factors such as mechanisms for eDNA deposition and persistence on wildflowers, and the ability of these primers to distinguish butterflies in complex environmental samples may be affecting the capability of detecting butterflies from wildflower‐associated eDNA. These issues provide a wealth of opportunity for future research.

Predicting whether a butterfly would be expected to leave eDNA behind on a wildflower could be a function of flower morphology, the way that the butterfly interacts with the flower (e.g., nectaring vs. resting), or the time spent in contact with a flower. The probability of eDNA sloughing off of a butterfly from saliva, skin cells, or excrement while it interacts with the flower could also be affected by butterfly age, sex, and morphology (James and Lohman 2023). Though butterflies frequently visit flowers to nectar or rest, their visits are often short and may not allow time for eDNA deposition. One study found that skipper butterflies (Hesperiidae) with medium to long proboscises spent an average of 6.5 s nectaring from short tubed flowers and 48 s from long tubed flowers (Bauder et al. 2015). Visits of *Speyeria mormonia* and *Plebejus melissa* in the contrived visit experiment totaled 50 s of resting time for the highest visit category (10 visits of 5 s each) and were not detected, so it could be that the average visit from a butterfly is not long enough to facilitate detection using the eDNA metabarcoding protocol described herein. Additional testing with controlled experiments and longer handling times will be needed to determine the limits of detection for butterflies using wildflower eDNA.

### 4.4 Detection of Small, Flower‐Associated Insects

Non‐Lepidopteran taxa detected were predominantly small‐bodied and often phytophagous, including Miridae, Tephritidae, and Chironomidae (Table 1). Miridae, or plant bugs are less than 10 mm in size and are known to feed on flowers (Pan et al. 2015; Etl et al. 2022). Tephrtidae (fruit flies) are less than 25 mm and the subfamily Tephritinae predominately specialize on *Asteraceae* flowers for larval development (Mazzon et al. 2010). More than half of the wildflower eDNA samples collected were of *Asteraceae* (168 of 298 flowers). Chironomidae, or midges, which are primarily associated with freshwater, may indicate detections originating from laboratory processing rather than true detections from the flowers. The two Lepidopteran families that were detected frequently with either primer set have larval stages associated with manipulating plant tissue, Tortricidae are commonly called leaf roller moths and Coleophoridae are commonly called casebearer moths or leaf miners. Traditional surveys did not include moth species, so we cannot confirm whether individuals of these families were present, though larvae from members of both families may feed on flowers and use flower tissue in case building (Powell and Opler 2009). Some vegetative tissue was also included with our flower samples with collections taken at the base of the stem. Physical specimens of only one of the most‐detected families, thrips (Thysanoptera), were observed on the flowers during sample preparation; they were found in the sample envelope after the flower had been frozen. However, it is possible that egg or larval stages of multiple insect species were present but not visible to the naked eye, which would result in more DNA material associated with the flower than trace DNA from flower contact alone. Life history traits, such as feeding or residing within flower tissues, may increase DNA deposition and detection probability. These patterns suggest that wildflower‐associated eDNA may be biased toward detecting small, abundant, or flower‐residing insects rather than short‐term flower visitors like butterflies. Thus, if a biodiversity survey had the goal of obtaining data on Lepidoptera taxa more broadly (e.g., butterflies, moths, and micromoths), a complementary approach of using aerial netting, light traps, and eDNA surveys could be a viable approach.

## 5 Conclusion

Wildflower‐associated eDNA metabarcoding shows promise for insect community studies, though results from this study demonstrate that much more concerted study will be needed to refine techniques. Further research on DNA deposition, persistence, primer biases, and optimal sampling strategies is required before making inferences on whole floral associated insect communities from eDNA metabarcoding. Controlled mesocosm and field experiments targeting specific taxa, alongside observational surveys, will be essential to develop reliable methods for assessing flower‐associated insect communities using eDNA. While pollinators remain of particular ecological and conservation interest, understanding the detection of smaller‐bodied flower‐associated insects will improve the interpretation of eDNA metabarcoding data and inform broader ecological studies.

## Author Contributions

**Cayley Faurot‐Daniels:** conceptualization (equal), data curation (equal), formal analysis (equal), investigation (lead), methodology (equal), validation (equal), visualization (lead), writing – original draft (lead), writing – review and editing (equal). **Diane M. Debinski:** conceptualization (equal), data curation (equal), formal analysis (equal), funding acquisition (lead), investigation (equal), methodology (equal), project administration (equal), supervision (equal), validation (equal), visualization (equal), writing – original draft (equal), writing – review and editing (equal). **Michelle L. Flenniken:** conceptualization (equal), data curation (equal), formal analysis (equal), funding acquisition (equal), investigation (equal), methodology (equal), project administration (equal), supervision (equal), validation (equal), visualization (equal), writing – original draft (equal), writing – review and editing (equal).

## Funding

This work was supported by the National Science Foundation Graduate Research Fellowship Program (Grant No. 2043105) and a Montana State University Research Expansion Grant.

## Conflicts of Interest

The authors declare no conflicts of interest.

## Supporting information


**Appendix S1:** ece374559‐sup‐0001‐AppendixA.xlsx.
Table A1. All wildflowers collected for eDNA analyses.
Table A2. Representation of Glacier National Park butterfly species in the COI Barcode of Life Database.
Table A3. Pooling of wildflower eDNA from samples without known butterfly visits.
Table A4. PCR primers used in the study.
Table A5. Description of final sequence library.
Table A6. Amplicon sequence variants (ASVs) that were removed from analysis as contaminants.
Table A7. ASVs that were removed from analysis by an additional sequence length filter.
Table A8. Raw sequences counts from Illumina NovaSeq and index hopping estimates.
Table A9: Environmental DNA (eDNA) sample sequence data.
Table A10. Sequence and quality control summary.
Table A11. Sequence data for positive control samples.
Table A12. Sequence comparison between Lepidopteran ASVs and regional COI records confirm target butterfly species were not identified from contrived visit experiment.
Table A13. Comparison of butterfly species detections from wildflower‐associated eDNA samples to contrived visitation, observed natural visitation, and known butterfly presence from traditional netting surveys.

**Appendix S2:** ece374559‐sup‐0002‐AppendixB.pdf.
Figure B1. *Cytochrome c oxidaseI*(COI) gene targets and samples summarized.
Figure B2. Nested eDNA metabarcoding and sequencing workflow.
Figure B3. COI primers align to positive control species *coI* genes.
Figure B4. Species identified in negative controls.
Figure B5. Unique amplicon sequence variants and taxa accumulate with additional technical replicates.
Figure B6. Butterfly detections differed between the two primer sets.

## Data Availability

Data from this project are available with a Creative Commons Zero designation (https://creativecommons.org/publicdomain/zero/1.0) in Dryad Digital Repository (https://datadryad.org), Dryad Dataset: Lepidoptera COI targeted eDNA from wildflowers, doi:10.5061/dryad.rv15dv4nv. Sequence data is available on the National Center for Biotechnology Information (NCBI) Sequence Read Archive (SRA) (BioProject PRJNA1422262).
