# Supplementary material for: Evaluating Metabarcoding of Wildflower Associated eDNA to Assess Butterfly Presence and Abundance
Source: Ecol Evol. 2026 Nov 11;16(11):e74559. doi: 10.1002/ece3.74559 (PMC13755521; doi:10.1002/ece3.74559)
Supplement: Supplementary file 1 [file ECE3-16-e74559-s002.pdf]

APPENDIX B – Supplemental Text and Figures for

Evaluating metabarcoding of wildflower associated eDNA to assess butterfly presence and abundance

This PDF file includes:

Supplementary Text
Figs B1-B6
References

Other supplementary materials for this manuscript include the following:

Appendix A: Supplemental Tables
Tables A1 – A10
Supplemental Files 1-6
Dryad Dataset: Lepidoptera COI targeted eDNA from wildflowers, doi:10.5061/dryad.rv15dv4nv)

Supplementary Text

DNA Extraction.
For all individual wildflower heads, DNA was washed off each flower and isolated using the Qiagen DNeasy Blood and Tissue Kit with a modified protocol as in previous studies extracting arthropod DNA from wildflowers (Thomsen & Sigsgaard, 2019; Harper et al., 2023). A buffer only negative control was added to each DNA extraction at the cell-lysis and incubation stage and carried throughout remaining analysis and sequencing. DNA concentration (ng/μL) was quantified for each sample using an Invitrogen Qubit 4.0 fluorometer and the 1x dsDNA High Sensitivity (HS) Assay Kit (Supplemental File 1).

Specifically, flowers were first transferred to sterile 50 mL tubes, taking care to orient flower heads towards the bottom of the tube. Buffer ATL and proteinase K (ProK, 600 m/AU) were added to the tubes in volumes specified by the size of the flower (small: 540 μL ATL and 60 μL ProK, medium: 900 μl ATL and 10 μL ProK, large: 1200 μl ATL and 130 μl Prok), vortexed 10 seconds, and placed in an incubated shaker at 56°C and 200 rpm for three hours. A buffer only negative control was also included at this step with 180 μL ATL and 20 μL ProK in a 2.0 mL tube included in incubation and all downstream steps through DNA extraction, PCR amplification and sequencing. Samples were vortexed for 10 seconds each hour. After incubation, the buffer was transferred to fresh 1.5 mL tubes and equal volumes of buffer AL and molecular grade ethanol (EtOH) were added. All volume was then spun through a spin column at 10,000 x g for one minute. Columns were washed with 600 μl each of AW1 and AW2 buffers and spun at 10,000 x g for one minute after each addition. Columns were then placed in fresh 1.5 mL tubes and DNA was eluted in two steps of 60 μL dH_2O with incubation at 37° C for 15 minutes, for a final volume of 120 μL soluble DNA.

PCR and Multiplexing
Two sub-regions of the standard 658-bp COI barcode were targeted using two different primer sets established for their ability to distinguish Lepidopteran species (Hajibabaei et al., 2006). MLepF1 (5'-GCTTTCCCACGAATAAATAATA-3') – LepR1 (5'-TAAACTTCTGGATGTCCAAAAAATCA-3'), referred to as 'Primer Set 1', targets a 407-bp amplicon ending on the 3' end of the barcode region and LepF1 (5'-ATTCAACCAATCATAAAGATATTGG-3') – MH-MR1 (5'-CCTGTTCCAGCTCCATTTTC-3'), referred to as 'Primer Set 2', targets a 311-bp amplicon beginning on the 5' end of the barcode region (Figure 3). The two primer regions overlap ~60 bp of the 658 bp COI barcode region.

DNA samples were amplified and multiplexed with a 2-step PCR nested metabarcoding method (Kitson et al., 2019; Guenay-Greunke et al., 2021). In the first PCR (PCR1), fusion primers (Integrated DNA Technologies) were used to add a unique 8-nucleotide sequence (internal index) for sample tagging, a 0-6-bp variable spacer to increase sequence heterogeneity, and an extension sequence to bind sequencing adapters in the second PCR (PCR2). Twelve forward and eight reverse primers with unique internal indices were used to tag a full 96-well PCR plate with unique combinations of indices on forward and reverse primers (Supplemental Table 3). However, 12 tag combinations were not used in the entire study to facilitate tracking of index hopping after sequencing, thus 84 PCR reactions could be performed on each PCR plate. Each PCR plate also

included two PCR positive controls (PosCtrl_noBG and PostCtrl_wBG), described above, and one no-template control (NTC), or dH_2O alone added as template.

The first PCR was conducted in a 25 µL reaction with 12.5 µL AmpliTaq Gold™ 360 Master Mix (Applied Biosystems), 1.25 µL each of 10 µM forward and reverse primers, 5 ng of DNA template, and dH_2O to 25 µL. Master mix, primers and dH_2O were added in a template-free clean room in a biosafety hood, and DNA template was added in a separate PCR room. Reactions were cycled on a Thermal Cycler with the following profile: 95° C for 10 minutes, 35 cycles of 95° C 10 seconds/46° C for 30 seconds/72° C for 1 minute, followed by five minutes at 72° C (Clarke et al., 2017).

PCR product concentration (ng/µL) was quantified for each sample using an Invitrogen Qubit 4.0 fluorometer and the 1x dsDNA High Sensitivity (HS) Assay Kit (Fisher Scientific). With each PCR reaction consisting of a unique combination of tags allowing product differentiation after sequencing, 30 ng of each reaction from the PCR plate was pooled in a 1.5 mL tube (PCR1 pools). Immediately after pooling, PCR1 pools were cleaned and size selected with SPRISelect (Beckman Coulter) magnetic beads. A 0.8x bead to sample ratio was used for a left-sided size selection of products above 200 bp. Briefly, 40 µL of SPRISelect beads were added to 50 µL of PCR1 pool, bound on a magnetic stand, washed with freshly diluted 85% EtOH, and the cleaned products were eluted with 50 µL dH_2O. The eluate was then cleaned a second time with the same protocol and the final cleaned PCR1 pool was eluted in 20 µL dH_2O.

A second PCR (PCR2) was used to add Illumina sequencing adapters and a second set of unique dual indices (Illumina Index) on each forward and reverse primer. Nine forward and reverse primers (IDT) with unique indices were used on the nine PCR1 pools (Supplemental Table 3). PCR2 consisted of 50 µL reactions with 25 µL AmpliTaq Gold™ 360 Master Mix, 1.0 µL each of 10 µM forward and reverse primers, 5 µL PCR1 pool template, and dH_2O to 50 µL. Master mix, primers and dH_2O were added in a template-free clean room underneath a biosafety hood and PCR1 pool template was added in a separate PCR room. Reactions were cycled on a Thermal Cycler with the following profile: 95° C for 10 minutes, 10 cycles of 95° C 10 seconds/55° C for 30 seconds/72° C for 1 minute, followed by five minutes at 72° C (Clarke et al., 2017).

PCR2 reactions, representing all samples pooled from a PCR plate, were cleaned with one round of 0.8x SPRISelect as described above. The size and concentration of cleaned PCR2 reactions, referred to as sub-libraries, were then obtained using an Agilent Bioanalyzer and the Agilent High Sensitivity DNA kit. Sub-libraries were pooled together in a final sequencing library to 10 µM, where each pre-library was diluted to a 5 µL stock at 10 µM, apart from pre-library 8 which was of too low concentration to obtain 10 µM and instead equal volume (5 µL) was added to the final pool (Supplemental Table 4). The final wildflower-associated eDNA sequencing library was quantified on the Agilent Bioanalyzer with the High Sensitivity DNA kit.

<u>High Throughput Sequencing and Bioinformatic Processing</u>

The wildflower-associated eDNA sequencing library was sent to the University of Illinois Roy J. Carver Biotechnology Center for 2 x 250-bp paired-end sequencing on the Illumina NovaSeq SP flowcell. Sequence data (806,792,426 reads) was de-multiplexed by Illumina indices (i.e., sub-libraries) by the University of Illinois. All further computation

efforts were performed on the Tempest High Performance Computing System, operated and supported by University Information Technology Research Cyberinfrastructure at Montana State University. First, sequences were further de-multiplexed by internal indices using Cutadapt v4.2 (Martin, 2011) with allowed errors set to 2 (0.25 error rate), resulting in sequences associated with individual samples. All possible combinations of indices were given in the Cutadapt search, including 12 primer index combinations that were not used in this study. Unused index combinations were used to derive (or calculate) an index hopping rate by comparing unexpected tag combinations to those that are expected (Schnell et al., 2015; Guenay-Greunke et al., 2021). Total sequences for all index combinations were found using SeqKit v2.7.0 (Shen et al., 2016) and proportions of unmatched and matched sequences were calculated in R v4.3.2 (R Core Team, 2023).

Demultiplexed files were renamed with their sample associated names in R and primers were removed using Cutadapt. All further bioinformatic processing was conducted using the DADA2 bioinformatic pipeline (Callahan et al., 2016) via the Targeted Amplicon Diversity Analysis (TADA) workflow implemented in Nextflow v22.10.8.5859 (Lennard et al., 2020) and Java v17.0, which includes quality filtering, sample denoising, read merging, and chimera removal. The TADA pipeline outputs amplicon sequence variants (ASVs), or sequences that vary by one or more nucleotides.

Taxonomic Assignment

Taxonomic assignment of unknown sequences obtained from eDNA relies on aligning unknown sequences to a reference database comprised of the COI barcode regions of the COXI gene. The Barcode of Life Database (BOLD) is the main repository for COI reference sequences with over 9 million vouchered specimens. For clarity, sequences associated with COI databases will be referred to as “records” and sequences obtained from wildflower eDNA will retain the term “sequences.”

COI reference databases

The full BOLD database was downloaded from the March 2023 versioned database and contained 9,237,843 records (BOLD, 2023). Several initial quality control and filtering steps were conducted from the Linux command line, including removing records associated with plants and markers other than COI, removing duplicated records, and removing records containing non-IUPAC characters (ACTG), which resulted in 8,287,203 records. Sequences associated with these records and accompanying taxonomy were imported into QIIME 2 v2024.2.0 (Bolyen et al., 2019) where sequences were culled further and dereplicated with RESCRIPt, a QIIME 2 plugin (Ii et al., 2021) leaving 3,747,990 records. Finally, records associated only with the United States and Canada were retained for a final database, hereafter referred to as ‘bold-full-USCAN’, representing 1,114,806 COI barcodes of animal species from North America (Supplemental Files 2 and 3).

Additionally, a North American butterfly specific COI database was downloaded by accessing the project name DS-USCANLEP which consists of 14,286 records representing the 835 butterfly species known from Canada and the United States (D’Ercole et al., 2021). A Glacier National Park butterfly specific database was created by filtering the DS-USCANLEP database using a species list created from butterflies

that were observed in the traditional butterfly diversity study (Chapter 1) or could be expected in the study area based on cross-referencing citizen science identifications (iNaturalist) with the Montana Field Guide (Montana Natural Heritage Program) relative density and recency plots pertaining to a species. The resulting list included 126 species of butterflies which corresponds to 6,345 records in the DS-USCANLEP database (Supplemental Table 5), hereafter referred to as DS-USCANLEP-GNPSps (Supplemental Files 4 and 5).

Taxonomic assignment of ASVs from each primer pair was conducted in QIIME 2 using a global alignment tool, VSEARCH (Rognes et al., 2016), where query sequences were aligned to the reference databases described above. The threshold for alignment of unknown sequences to the reference database was set to 95% similarity. This value was based from COI barcode sequence alignments from all records in the DS-USCANLEP-GNPSps database. The FASTA formatted sequences associated with these records were imported into Geneious Prime (2023.0.1) and aligned using Muscle 5.1 with the Super5 alignment algorithm (Edgar, 2004). All 6,345 records from the DS-USCANLEP-GNPSps database were aligned and had an average of 87.1% similarity, indicating the VSEARCH default alignment threshold of 80% would be insufficient for elucidating target butterfly species-specific differences. Several species-specific alignments were done where all records associated with a species were aligned. Records of three of the species with the highest representation in the database and representing different butterfly families were aligned; 279 records of *Boloria chariclea* (Nymphalidae) shared 99.3% similarity, 242 records of *Glaucopsyche lygdamus* (Lycaenidae) shared 98.2% similarity, and 210 records of *Burnsius communis* (Hesperiidae) shared 99.9% similarity. The two species used in manipulated visits, *Speyeria mormonia* (Nymphalidae) and *Plebejus melissa* (Lycaenidae), were 99.1% (95 records) and 99.3% (41 records) similar, respectively. Records from *Plebejus idas* which is closely related to *Plebejus melissa* and difficult to distinguish in the field shared 96.3% similarity (122 records) and the 93 records of the butterfly species used in the positive control, *Pieris rapae* (Pieridae), shared 98.7% similarity. Species with fewer records in the database followed a similar pattern, where 5 records of *Pterourus multicaudata* (Papilionidae) shared 99.8%, 11 records of *Tharsalea nivalis* (Lycaenidae) shared 99.9% and 15 records of *Euchloe olympia* (Pieridae) shared 97.3% similarity. Because the within-species similarity varied from 96.3% to 99.9% and the percent similarity between butterfly species expected in Glacier was 87.1%, it was determined that a 95% similarity threshold would best facilitate assignment of unknown sequences in the wildflower-eDNA library described herein.

The VSEARCH global alignment tool was implemented in QIIME 2 using the q2-feature-classifier (Bokulich et al., 2018) and classify-consensus-vsearch (Rognes et al., 2016) plugins where after alignment of query sequences to the reference database, a consensus taxonomy is assigned by selecting the top hit among a maximum of accepted hits (‘maxaccepts’ set to 100) of which more than 50% share consensus with the top hit (‘minconsensus’, default 0.51). Output tables of taxonomic assignment results include the ASV feature identifier, the consensus taxonomy and the consensus score. These tables were imported into R v4.3.2 for the remainder of analysis.

Final cleaning and filtering steps

Several quality control and filtering steps were applied to sequences during the TADA bioinformatic pipeline and two more filtering steps, filtering out likely contaminants and applying a sequence length filter, were applied before analysis. There were 7,466 amplicon sequence variants (ASVs), or sequences that differ by one or more nucleotides, output from the TADA bioinformatic pipeline for Primer Set 1 (MLepF1/MHMR1) and 2,681 ASVs for Primer Set 2 (LepF1/MHMR1). Subsequent filtering and analyses were conducted using R v4.3.2 (R Core Team, 2023) and packages phyloseq v1.46.0 (McMurdie & Holmes, 2013) and decontam v1.22.0 (Davis et al., 2018). Data organization (or sorting) was done using phyloseq and tidyr/dplyr (v1.3.1/v1.1.4). The ASV tables, sample metadata and taxonomy files were merged using phyloseq. Likely contaminants were removed using the 'frequency' function of the decontam package that considers sequence abundance and DNA concentration of input template to recognize likely contaminates using the property that contaminant sequence abundance will scale linearly with the concentration of DNA, while true sequence abundance will be independent of DNA concentration (Davis et al., 2018). Dilution experiment samples (PL9) were analyzed separately and not filtered using decontam since DNA concentrations varied by design and would introduce bias using a frequency-based definition of contaminants. From all remaining samples (PL1 – PL8), twenty-five contaminants were identified by decontam for Primer Set 1 data and nine contaminants were identified for Primer Set 2 (Supplemental Table 6). The contaminant ASVs, as well as any ASVs with less than five associated sequences, were removed using phyloseq. An additional length filter was applied to remove sequences that were 100 bp shorter than the expected size for each primer pair (i.e., less than 310-bp for Primer Set 1 and less than 210-bp for Primer Set 2, Supplemental Table 7). This resulted in 122 fewer ASVs for the Primer Set 1 and 54 fewer ASVs for Primer Set 2. Finally, ASVs identified to any of the five positive control insect species were removed from wildflower-associated samples after finding two positive control species, *Phymata americana* and *Pieris rapae*, in negative control samples and in 136 of 672 (20.3%) and 142 of 672 (21.1%) of wildflower-associated samples, respectively. The final ASV and taxonomy table contained 8,147 ASVs between both primer sets (Supplemental File 6).

Supplemental Figures

A)

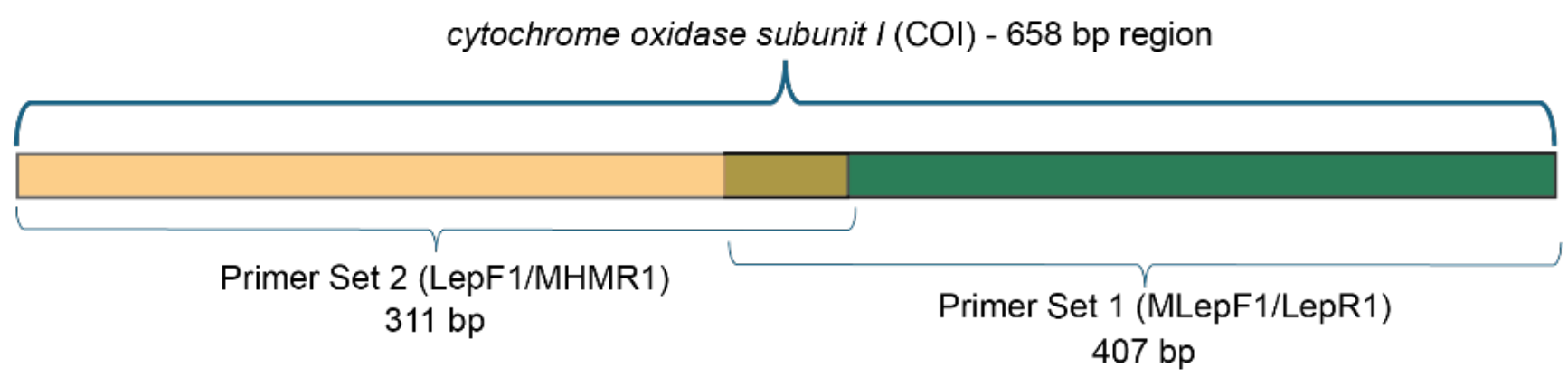

B) priority samples
3 technical replicates

Contrived Visit (CV)

# of visits	Speyeria (N)	Plebejus (N)
1	5	5
5	5	5
10	3	5
genus total	13	15
	0 visit (control)	3
	**experiment total**	**31**

Known Visit (KV)

flower genus	butterfly genus	# flower butterfly pair
Erigeron	*Speyeria*	3
Symphyotrichum	*Tharsalea*	3
Senecio	*Pontia*	3
Erigeron	*Phyciodes*	2
Erigeron	*Colias*	2
Erigeron	*Thymelicus*	2
Solidago	*Tharsalea*	2
Arnica	*Boloria*	1
Arnica	*Phyciodes*	1
Erigeron	*Cercyonis*	1
Erigeron	*Pontia*	1
Erigeron	*Aglais*	1
Erigeron	*Hesperia*	1
Senecio	*Aglais*	1
Senecio	Colias	1
	**total**	**25**

Unknown Pool (UNP)

flower genus	# pools	# flowers represented
Arnica	9	28
Erigeron	6	19
Achillea	3	10
Solidago	3	8
Potentilla	2	7
Senecio	2	5
**total**	**25**	**77**

C) remaining samples
1 replicate

Known Visit (KV)

flower genus	butterfly genus	N flower-butterly pair
Arnica	*Tharsalea*	1
Arnica	*Speyeria*	1
Arnica	*Thymelicus*	1
Erigeron	*Speyeria*	4
Erigeron	*Phyciodes*	1
Erigeron	*Tharsalea*	1
Erigeron	*Pontia*	1
Symphyotrichum	*Tharsalea*	7
Geranium	*Plebejus*	1
	**total**	**18**

Unknown Pool (UNP)

flower genus	# pools	# flowers represented
Dasiphora	6	18
Eriogonum	9	29
Packera	3	10
Geranium	9	28
Senecio	3	10
Drymocallis	3	10
Achillea	3	10
Potentilla	3	10
Arnica	6	20
**total**	**45**	**145**

Negative Controls

type	N
field negative	9
buffer negative	14

D) positive control dilutions
1 replicate

Positive Control Species	Stocks
Phymata americana	5.0 ng/ul
Pieris rapae	2.5 ng/ul
Coccinella transversoguttata	1.25 ng/ul
Apis mellifera	0.625 ng/ul
Bombus rufocinctus	

Dilution	Background (BG)
5.0 ng	dH20
2.5 ng	dH20
1.25 ng	dH20
0.625 ng	dH20
5.0 ng	mixed floral
2.5 ng	mixed floral
1.25 ng	mixed floral
0.625 ng	mixed floral
2.5 ng	*Arnica* floral
2.5ng	*Erigeron* floral
2.5 ng	*Potentilla* floral
2.5 ng	*Senecio* floral
2.5 ng	*Solidago* floral

Figure B1. *Cytochrome c oxidase I* (COI) gene targets and samples summarized. A) Two regions within the 658-base pair (bp) “barcode region” of the mitochondrial encoded *cytochrome oxidase subunit I* (COI) gene were targeted for amplification using Primer Set 1 (MLepF1/LepR1) or Primer Set 2 (LepF1/MHMR1). B) Select wildflower associated eDNA samples were amplified in triplicate using each primer set.
C) Additional samples were amplified in one replicate with each primer set.
D) One replicate of positive control dilution tests was performed with each primer set.

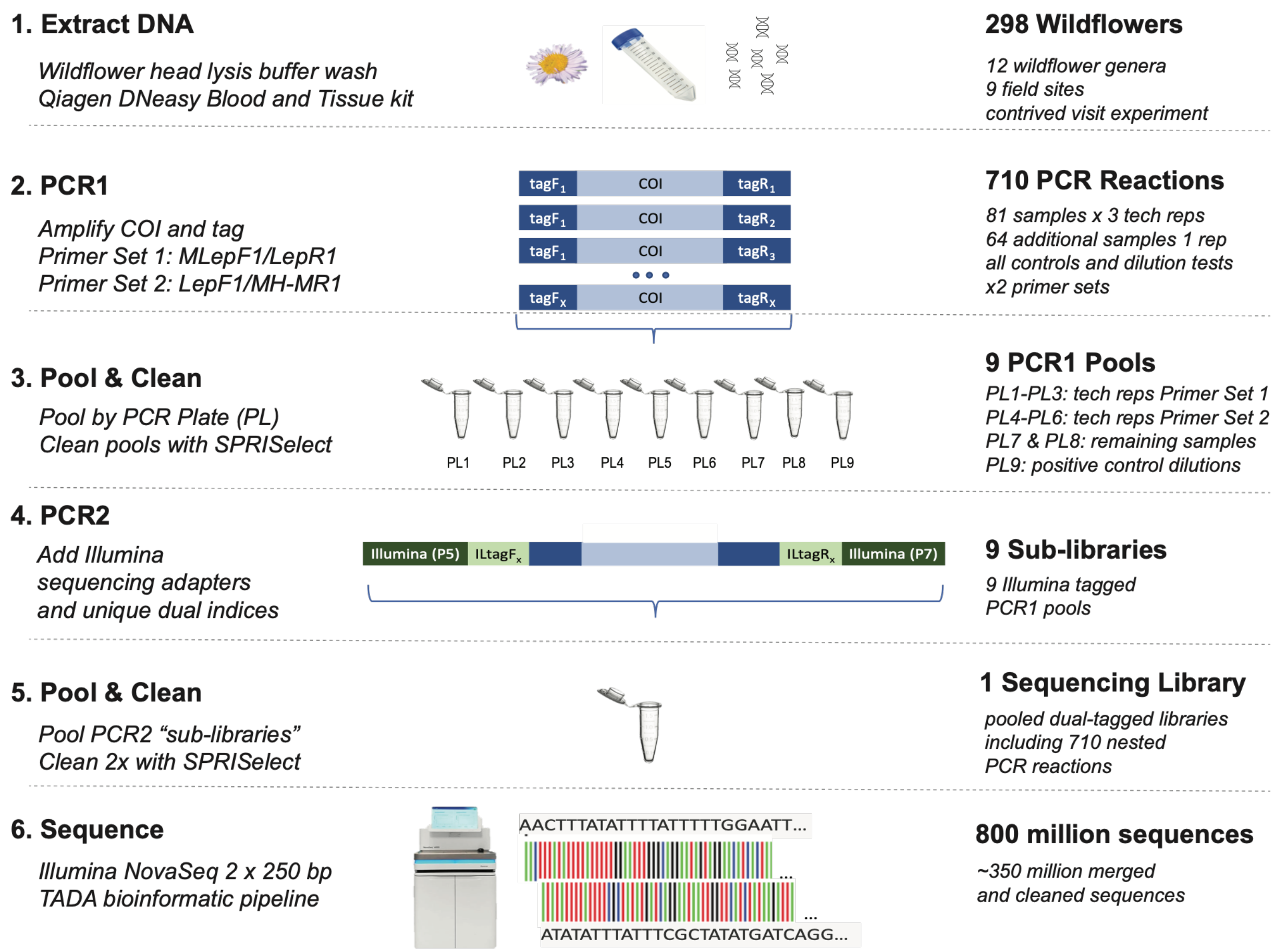

Figure B2. Nested eDNA metabarcoding and sequencing workflow.
DNA obtained from flower and control samples was prepared for high throughput sequencing (Illumina NovSeq) by amplifying two *cytochrome c oxidase I* (COI) gene regions (PCR1). The PCR1 products were generated with unique tag combinations, such that the amplicons generated on each 96-well plate (PL) could be pooled for a second PCR amplification step (PCR2) that added Illumina sequencing adapters and unique dual indexes (5' and 3' tags). The nine PCR2 sub-libraries were pooled into one library for sequencing with Illumina NovaSeq that resulted in ~ 800 million paired-end sequences. After bioinformatic processing using the targeted amplicon diversity analysis (TADA) pipeline, including quality filtering, read merging, denoising, and chimera removal, approximately 350 million sequences were retained for analyses.

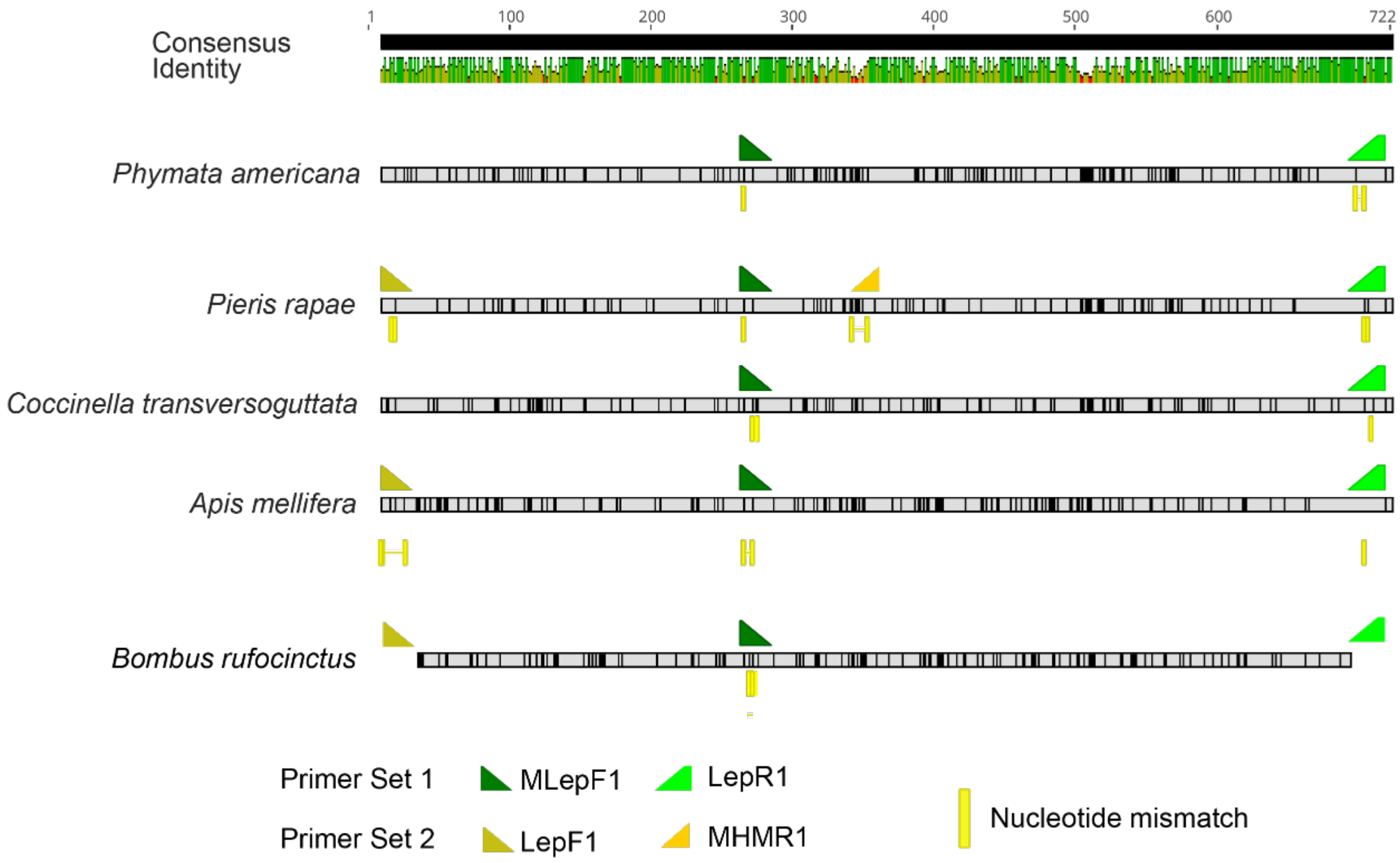

Figure B3. COI primers align to positive control species *col* genes.
Positive control sample sequencing libraries were generated using both primer sets which amplified a mixed species positive control of equal amounts of DNA from five insect species: one plant bug (*Phymata americana*), one butterfly (*Pieris rapae*), one beetle (*Coccinella transversoguttata*), and two bees (*Bombus rufocinctus* and *Apis mellifera*). Nucleotide alignments indicate that Primer Set 1 should amplify DNA from all five species since the primers have two or fewer mismatches with the target sequence. In contrast, Primer Set 2, which is more specific, was only expected to amplify *Pieris rapae* DNA. The primers LepF1 and LepR1 were used to create the COI reference sequence for *Bombus rufocinctus,* accessed from the Barcode of Life Database (BOLD), but longer sequences of the *col* gene from *Bombus rufocinctus* or closely related species were not available.

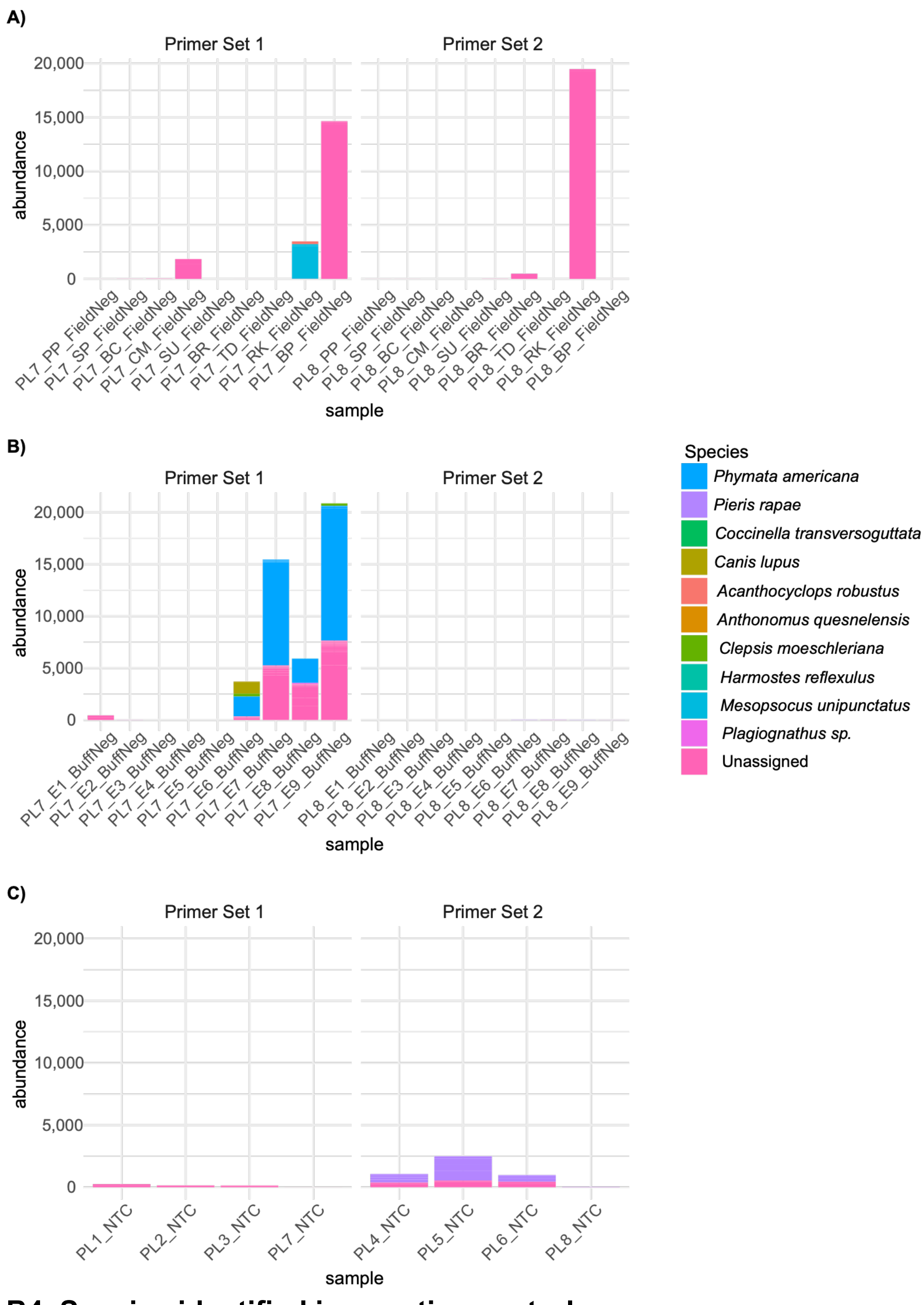

Figure B4. Species identified in negative controls.
A) Each wildflower collection event included a field negative control where a sterile gauze pad was opened and handled with sampling equipment, B) a buffer only negative control for each DNA extraction, and C) a no template controls (NTC) on each PCR plate. All negative controls were sequenced with both primer sets. *Phymata americana* was the most abundant species detected in negative control samples using Primer Set 1 and *Pieris* was identified in no template controls of plates 4-6 (Primer Set 2), both species used in positive controls.

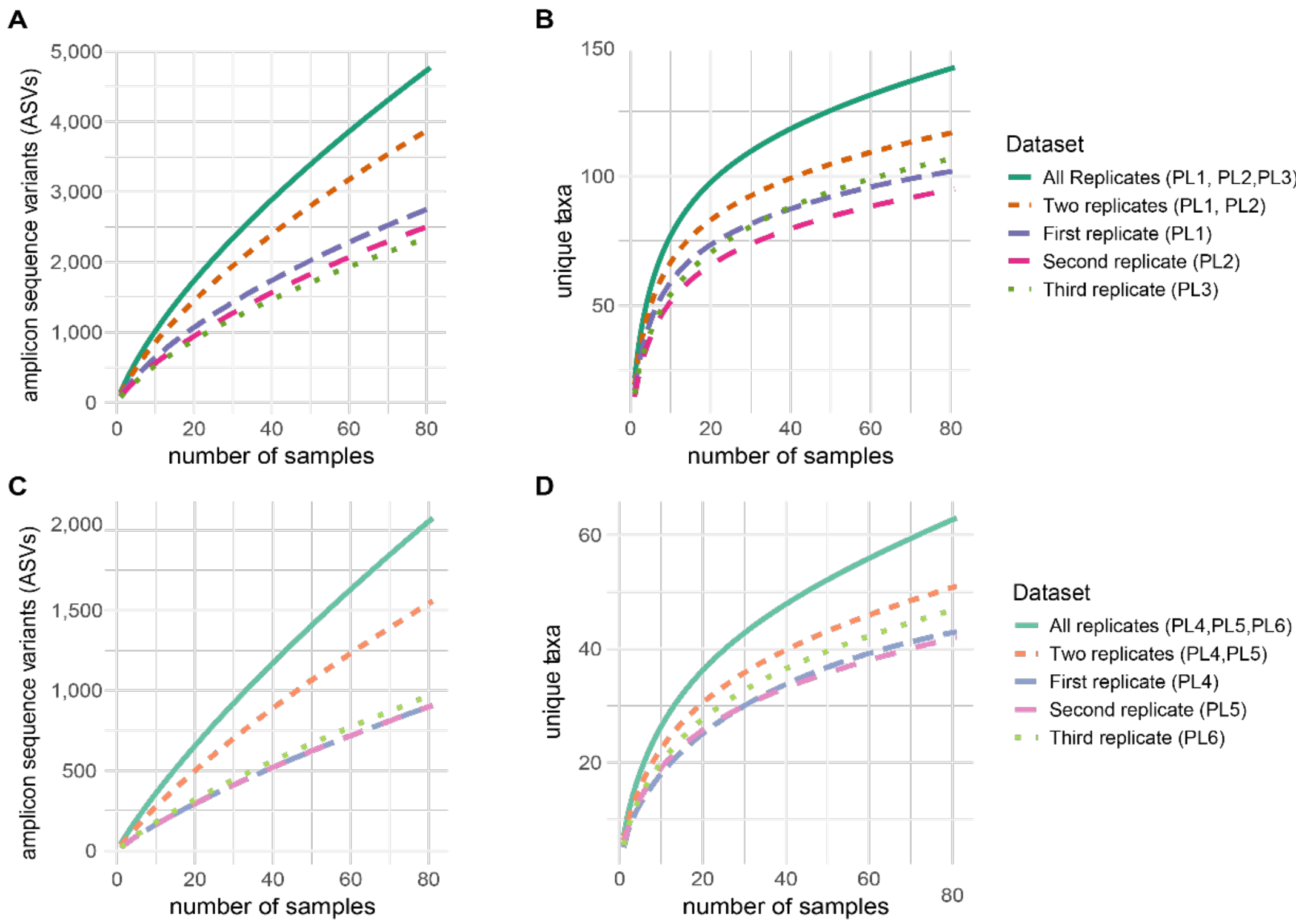

Figure B5. Unique amplicon sequence variants and taxa accumulate with additional technical replicates.

The accumulation of unique amplicon sequence variants (ASVs) and unique taxa identified from the ASVs for three technical replicates of 81 samples, A) The number of ASVs, and B) unique taxa identified with Primer Set 1, illustrating accumulation of additional identifications with additional replicates (i.e., plates 1-3). C) The number of ASVs and D) unique taxa identified with Primer Set 2, illustrating accumulation of additional identifications with additional replicates (i.e., plates 4-6).

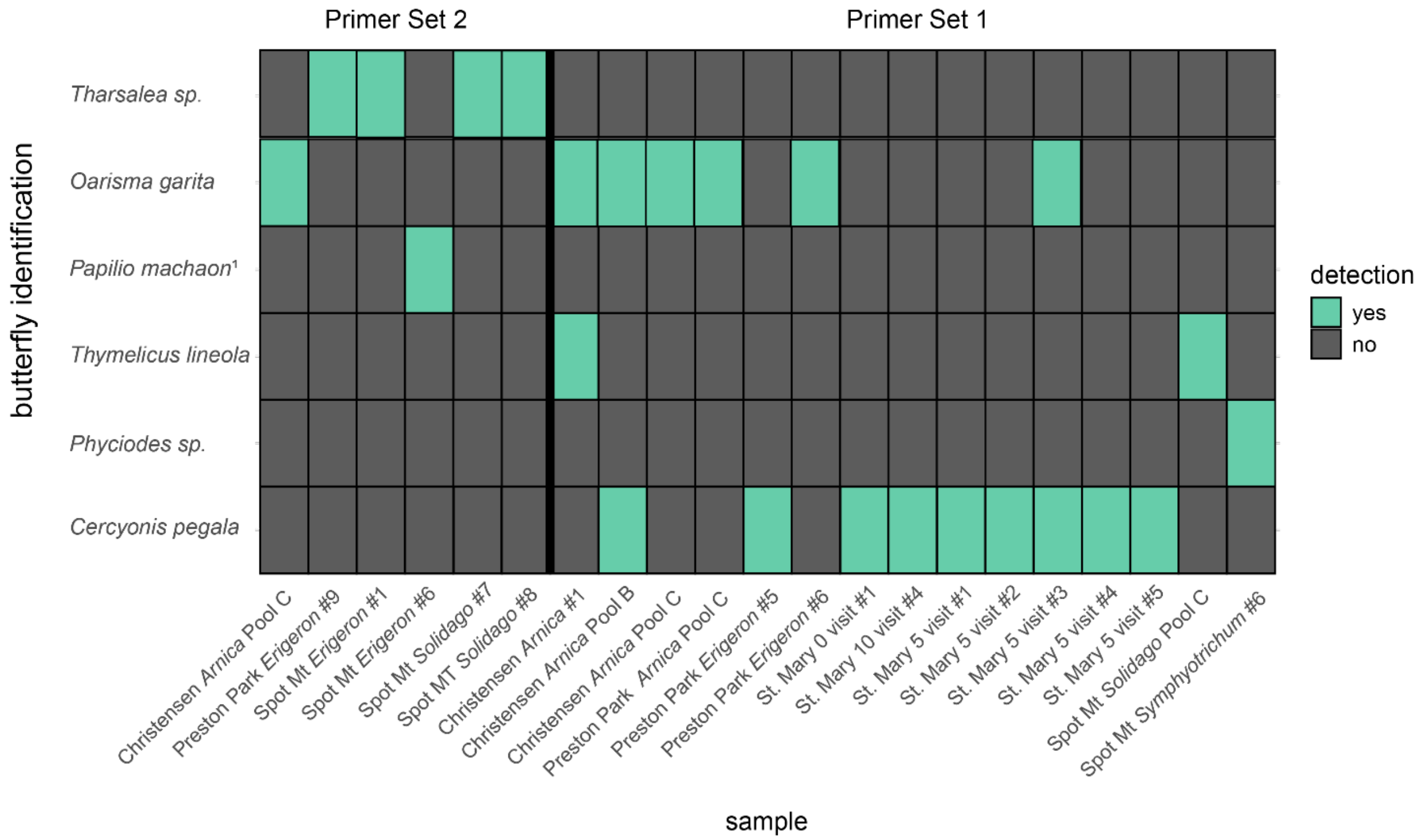

Figure B6. Butterfly detections differed between the two primer sets.
DNA from six butterfly taxa, four to species and two to genus, were identified a total of 24 times across 21 samples from four sites. Only one species, *Oarisma garita,* was identified with both primer sets. Green tiles indicate species detected and grey tiles absence. The vertical black bar divides detections from the two primer sets.

Supplemental Tables included in Appendix A (separate file)

Table A1. **All wildflowers collected for eDNA analyses.**

Table A2. Representation of Glacier National Park butterfly species in the COI Barcode of Life Database.

Table A3. Pooling of wildflower eDNA from samples without known butterfly visits.

Table A4. PCR primers used in the study.

Table A5. Description of final sequence library.

Table A6. Amplicon sequence variants (ASVs) that were removed from analysis as contaminants.

Table A7. ASVs that were removed from analysis by an additional sequence length filter.

Table A8. Raw sequences counts from Illumina NovaSeq and index hopping estimates.

Table A9. Sequence and quality control summary.

Table A10. Sequence comparison between Lepidopteran ASVs and regional COI records confirm target butterfly species were not identified from contrived visit experiment.

Data files included on Dryad
Lepidoptera COI targeted eDNA from wildflowers, doi:10.5061/dryad.rv15dv4nv

Supplemental File 1. Metadata for all wildflower-associated eDNA samples.

Supplemental File 2. Taxonomic information for regional COI database (bold-full-USCAN).

Supplemental File 3. Fasta formatted sequences for the regional COI database (bold-full-USCAN).

Supplemental File 4. Taxonomic information for the Glacier specific butterfly COI database (DS-USCANLEP-GNPSps).

Supplemental File 5. Fasta formatted sequences for the Glacier specific butterfly COI database (DS-USCANLEP-GNPSps).

Supplemental File 6. Final ASV-taxonomy table used in analyses.

References

Bokulich, N. A., Kaehler, B. D., Rideout, J. R., Dillon, M., Bolyen, E., Knight, R., Huttley, G. A., & Caporaso, J. G. (2018). Optimizing taxonomic classification of marker-gene amplicon sequences with QIIME 2's q2-feature-classifier plugin. *Microbiome*, *6*(1), 90. https://doi.org/10.1186/s40168-018-0470-z

BOLD, I. D. B. (2023). *BOLD DNA Barcode Reference Library 31-MAR-2023*. https://doi.org/10.5883/DP-BOLD_Public.31-Mar-2023

Bolyen, E., Rideout, J. R., Dillon, M. R., Bokulich, N. A., Abnet, C. C., Al-Ghalith, G. A., Alexander, H., Alm, E. J., Arumugam, M., Asnicar, F., Bai, Y., Bisanz, J. E., Bittinger, K., Brejnrod, A., Brislawn, C. J., Brown, C. T., Callahan, B. J., Caraballo-Rodríguez, A. M., Chase, J., … Caporaso, J. G. (2019). Reproducible, interactive, scalable and extensible microbiome data science using QIIME 2. *Nature Biotechnology*, *37*(8), 852–857. https://doi.org/10.1038/s41587-019-0209-9

Callahan, B. J., McMurdie, P. J., Rosen, M. J., Han, A. W., Johnson, A. J. A., & Holmes, S. P. (2016). DADA2: High-resolution sample inference from Illumina amplicon data. *Nature Methods*, *13*(7), Article 7. https://doi.org/10.1038/nmeth.3869

Clarke, L. J., Beard, J. M., Swadling, K. M., & Deagle, B. E. (2017). Effect of marker choice and thermal cycling protocol on zooplankton DNA metabarcoding studies. *Ecology and Evolution*, *7*(3), 873–883. https://doi.org/10.1002/ece3.2667

Davis, N. M., Proctor, D. M., Holmes, S. P., Relman, D. A., & Callahan, B. J. (2018). Simple statistical identification and removal of contaminant sequences in marker- gene and metagenomics data. *Microbiome*, *6*(1), 226. https://doi.org/10.1186/s40168-018-0605-2

D'Ercole, J., Dincă, V., Opler, P. A., Kondla, N., Schmidt, C., Phillips, J. D., Robbins, R., Burns, J. M., Miller, S. E., Grishin, N., Zakharov, E. V., DeWaard, J. R., Ratnasingham, S., & Hebert, P. D. N. (2021). A DNA barcode library for the butterflies of North America. *PeerJ*, *9*, e11157. https://doi.org/10.7717/peerj.11157

Edgar, R. C. (2004). MUSCLE: Multiple sequence alignment with high accuracy and high throughput. *Nucleic Acids Research*, *32*(5), 1792–1797. https://doi.org/10.1093/nar/gkh340

Guenay-Greunke, Y., Bohan, D. A., Traugott, M., & Wallinger, C. (2021). Handling of targeted amplicon sequencing data focusing on index hopping and demultiplexing using a nested metabarcoding approach in ecology. *Scientific Reports*, *11*(1), Article 1. https://doi.org/10.1038/s41598-021-98018-4

Hajibabaei, M., Janzen, D. H., Burns, J. M., Hallwachs, W., & Hebert, P. D. N. (2006). DNA barcodes distinguish species of tropical Lepidoptera. *Proceedings of the National Academy of Sciences*, *103*(4), 968–971. https://doi.org/10.1073/pnas.0510466103

Harper, L. R., Niemiller, M. L., Benito, J. B., Paddock, L. E., Knittle, E., Molano-Flores, B., & Davis, M. A. (2023). BeeDNA: Microfluidic environmental DNA metabarcoding as a tool for connecting plant and pollinator communities. *Environmental DNA*, *5*(1), 191–211. https://doi.org/10.1002/edn3.370

Ii, M. S. R., O'Rourke, D. R., Kaehler, B. D., Ziemski, M., Dillon, M. R., Foster, J. T., & Bokulich, N. A. (2021). RESCRIPt: Reproducible sequence taxonomy reference database management. *PLOS Computational Biology*, *17*(11), e1009581.https://doi.org/10.1371/journal.pcbi.1009581

Kitson, J. J. N., Hahn, C., Sands, R. J., Straw, N. A., Evans, D. M., & Lunt, D. H. (2019). Detecting host–parasitoid interactions in an invasive Lepidopteran using nested tagging DNA metabarcoding. *Molecular Ecology*, *28*(2), 471–483. https://doi.org/10.1111/mec.14518

Lennard, K., Fields, C., Botha, G., Holmes, J., Di Tommaso, P., & Clark, L. (2020). *TADA: Beta v0.5 release* (Version 0.5-beta). https://doi.org/10.5281/zenodo.4208836

Martin, M. (2011). Cutadapt removes adapter sequences from high-throughput sequencing reads. *EMBnet.Journal*, *17*(1), Article 1. https://doi.org/10.14806/ej.17.1.200

McMurdie, P. J., & Holmes, S. (2013). phyloseq: An R Package for Reproducible Interactive Analysis and Graphics of Microbiome Census Data. *PLOS ONE*, *8*(4), e61217. https://doi.org/10.1371/journal.pone.0061217

R Core Team. (2023). *R: A Language and Environment for Statistical Computing*. R Foundation for Statistical Computing. https://www.R-project.org/

Rognes, T., Flouri, T., Nichols, B., Quince, C., & Mahé, F. (2016). VSEARCH: a versatile open source tool for metagenomics. *PeerJ*, *4*, e2584. https://doi.org/10.7717/peerj.2584

Schnell, I. B., Bohmann, K., & Gilbert, M. T. P. (2015). Tag jumps illuminated – reducing sequence-to-sample misidentifications in metabarcoding studies. *Molecular Ecology Resources*, *15*(6), 1289–1303. https://doi.org/10.1111/1755-0998.12402

Shen, W., Le, S., Li, Y., & Hu, F. (2016). SeqKit: A Cross-Platform and Ultrafast Toolkit for FASTA/Q File Manipulation. *PLOS ONE*, *11*(10), e0163962. https://doi.org/10.1371/journal.pone.0163962

Thomsen, P. F., & Sigsgaard, E. E. (2019). Environmental DNA metabarcoding of wildflowers reveals diverse communities of terrestrial arthropods. *Ecology and Evolution*, *9*(4), 1665–1679. https://doi.org/10.1002/ece3.4809
